# Histomorphological and Molecular Features of Colonic Large-Cell Neuroendocrine Carcinoma in a Patient With Familial Adenomatous Polyposis: A Case Report and Review of Literature

**DOI:** 10.1177/10668969251346939

**Published:** 2025-06-10

**Authors:** Mohamed Moustafa, Jing Xu, Hua Wang, Lan Peng, Zhikai Chi

**Affiliations:** 1 Department of Pathology, 12334 The University of Texas Southwestern Medical Center, Dallas, TX, USA

**Keywords:** colon, cecum, large-cell neuroendocrine carcinoma, familial adenomatous polyposis

## Abstract

Colorectal large-cell neuroendocrine carcinoma, a rare and aggressive type of cancer, accounts for <0.6% of all colorectal cancers. Neuroendocrine carcinomas are associated with hereditary conditions such as Lynch syndrome; however, their co-occurrence with familial adenomatous polyposis (FAP) is poorly documented. To date, only 1 patient of colorectal neuroendocrine carcinoma in a patient with FAP has been reported. This report presents a patient with FAP. Large-cell neuroendocrine carcinoma with lymph node metastasis was discovered during right colectomy. Histopathological and immunohistochemical assessments confirmed neuroendocrine differentiation with a high Ki-67 index (>90%). Genetic analysis revealed a pathogenic germline *APC* mutation and somatic alterations in *APC*, *TP53, RB1, PALB2, MAP3K1, NTRK3,* and *KRAS*. Adjuvant chemotherapy commenced postoperatively. No evidence of recurrence was observed for 18 months postoperatively. This case report highlights the rare presentation of colorectal large-cell neuroendocrine carcinoma in a patient with FAP, thereby contributing to the limited literature on this association. *APC* mutations have been characterized in adenomatous polyposis and colorectal adenocarcinomas; however, their role in the pathogenesis of neuroendocrine carcinoma remains unclear. Additional mutations of *TP53*, *RB1*, *PALB2*, *MAP3K1*, *NTRK3*, and *KRAS* suggest a unique molecular profile that may contribute to the development of neuroendocrine carcinoma in patients with FAP. This is the second reported patient of colorectal large-cell neuroendocrine carcinoma in a patient with FAP. Further studies must be conducted to elucidate the role of *APC* mutations in the pathogenesis of neuroendocrine tumorigenesis.

## Introduction

Neuroendocrine carcinoma is a rare and highly aggressive subset of neuroendocrine neoplasms. Neuroendocrine neoplasms predominantly develops in the lungs and gastrointestinal tract; however, it can develop in nearly any organ.^1^ The World Health Organization classifies colonic neuroendocrine neoplasm as epithelial neoplasms with neuroendocrine differentiation that can be divided into well-differentiated neuroendocrine tumors and poorly-differentiated neuroendocrine carcinoma.^2^ The cells of the neuroendocrine neoplasms of well-differentiated neuroendocrine tumor retain the structural features of normal neuroendocrine cells for hormone synthesis and secretion. Neuroendocrine carcinoma are high-grade malignant neoplasms with cells that express little or no chromogranin A, somatostatin receptors, or hormones; however, high expression of INSM1 and synaptophysin has been observed in neuroendocrine carcinoma. Neuroendocrine carcinoma is poorly differentiated and high-grade by definition. Notably, it exhibits high proliferative activity and can be classified as small cell neuroendocrine carcinoma and large cell neuroendocrine carcinoma based on the morphology.^3^ A high mitotic rate of >20 per 10 high-power fields and a Ki-67 index of >20% have been observed in neuroendocrine carcinoma. Small cell neuroendocrine carcinoma is smaller than three resting lymphocytes in >90% of the tumor cells. The morphological features of small cell neuroendocrine carcinoma include scanty cytoplasm, ill-defined borders, finely granular nuclear chromatin, inconspicuous nucleoli, nuclear molding, and a sheet-like or diffuse architecture. In contrast, large-cell neuroendocrine carcinoma exhibits an organoid or diffuse growth pattern with frequent necrosis. The tumor cells are large with irregular nuclei, prominent nucleoli, and distinct cell borders.^3^ Colorectal neuroendocrine carcinoma, irrespective of the morphology, is exceedingly rare, accounting for only 1 to 2 patients per million annually,^4^ constituting approximately 0.6% of colorectal cancers.^5^

Key genetic alterations drive the aggressive nature of neuroendocrine carcinoma. The most frequent mutations involve *TP53* and *RB1*, which play crucial roles in tumor suppression and regulation of the cell cycle. *TP53* mutations, which are nearly ubiquitous in neuroendocrine carcinoma, result in a loss of genomic stability. In contrast, *RB1* inactivation, which is particularly common in small cell neuroendocrine carcinoma, contributes to uncontrolled cell proliferation. Additionally, *KRAS* mutations are frequently observed in gastrointestinal and pancreatic neuroendocrine carcinoma. These mutations often co-occur with *TP53* mutations. Amplifications in the *MYC* family (*MYC, MYCN*, and *MYCL*) are other oncogenic drivers associated with increased tumor aggressiveness and poor prognosis. Mutations in the PIK3CA and PTEN pathways are occasionally observed in gastrointestinal neuroendocrine carcinoma. These mutations promote tumor growth and survival through aberrant PI3K/AKT signaling.^6^

Lynch syndrome, which is characterized by mutations in mismatch repair genes, coexists in approximately 15% of patients with colorectal neuroendocrine carcinoma.^7^ However, the association between FAP and neuroendocrine carcinoma is poorly understood. Although *APC* mutations have been detected in patients with neuroendocrine carcinoma, co-occurrence of FAP with neuroendocrine carcinoma is exceedingly rare.^8^ This case report aims to expand the current understanding of neuroendocrine carcinoma in patients with FAP, with potential implications for its diagnosis and management.

## Patient Presentation

A woman in her late 40s presented to the emergency department with abdominal pain, tachycardia, and leukocytosis. Computed tomography revealed the presence of a mass in the right lower quadrant of the abdomen near the cecum with possible perforation. In addition, enlarged lymph nodes were observed in the ileocolic, para-aortic, and gastrohepatic regions. Colonoscopy revealed a fungating, partially obstructed, circumferential mass in the cecum with >100 colonic polyps. Esophagogastroduodenoscopy also revealed >100 gastric polyps. Germline genetic test detected a heterozygous, pathogenic germline mutation in the *APC* (C1270*), which indicated familial adenomatous polyposis (FAP). Bowel rest, intravenous antibiotics, and fluid resuscitation were prescribed initially. However, the patient returned with fever and pain in the right lower quadrant following discharge and was diagnosed with an abdominal abscess. Presentation of fever and worsening abdominal led to the decision to perform an emergency right colectomy with diverting loop ileostomy to relieve obstruction.

**Figure 1.**
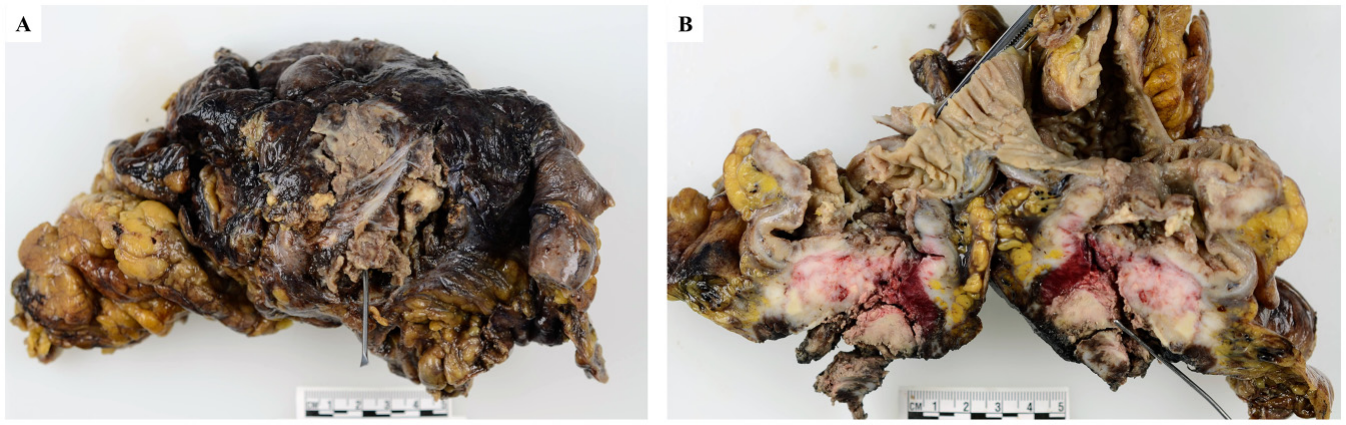
Gross examination. The tumor was centered in the cecum, with a full-thickness perforation. A, from outside; B, bisected.

Surgical resection revealed a circumferential tumor measuring 8.0 × 4.5 cm extending from the cecum to the terminal ileum involving the appendix. The primary tumor, with a full-thickness perforation, was centered in the cecum (Figure 1). The tumor was located 8.0 cm and 14.0 cm from the proximal and distal margins, respectively, with abutting the radial and mesenteric margins. Numerous mucosal polyps (>100) were observed in the remaining large bowel mucosa, consistent with the history of FAP.

**Figure 2.**
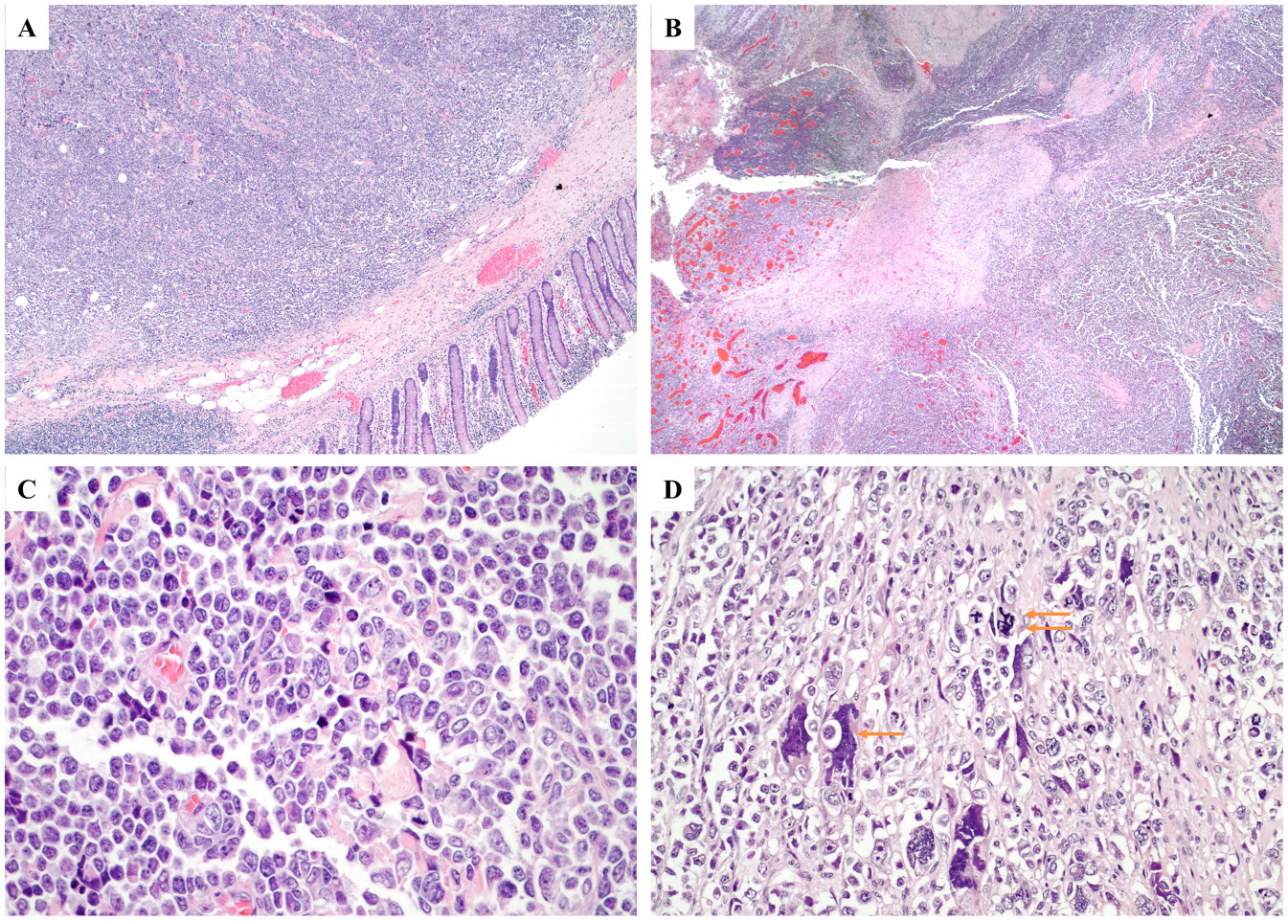
Microscopic examination. (A) Sheets of tumor cells with colonic mucosa (hematoxylin and eosin [H&E], 20×). (B) Tumor necrosis (H&E, 20×). (C) Large tumor cells with pleomorphic nuclei, coarse chromatin, and prominent nucleoli (H&E, 200×). (D) Large, atypical tumor cells (single arrow) and atypical mitotic figures (double arrows) (H&E, 200×).

**Figure 3.**
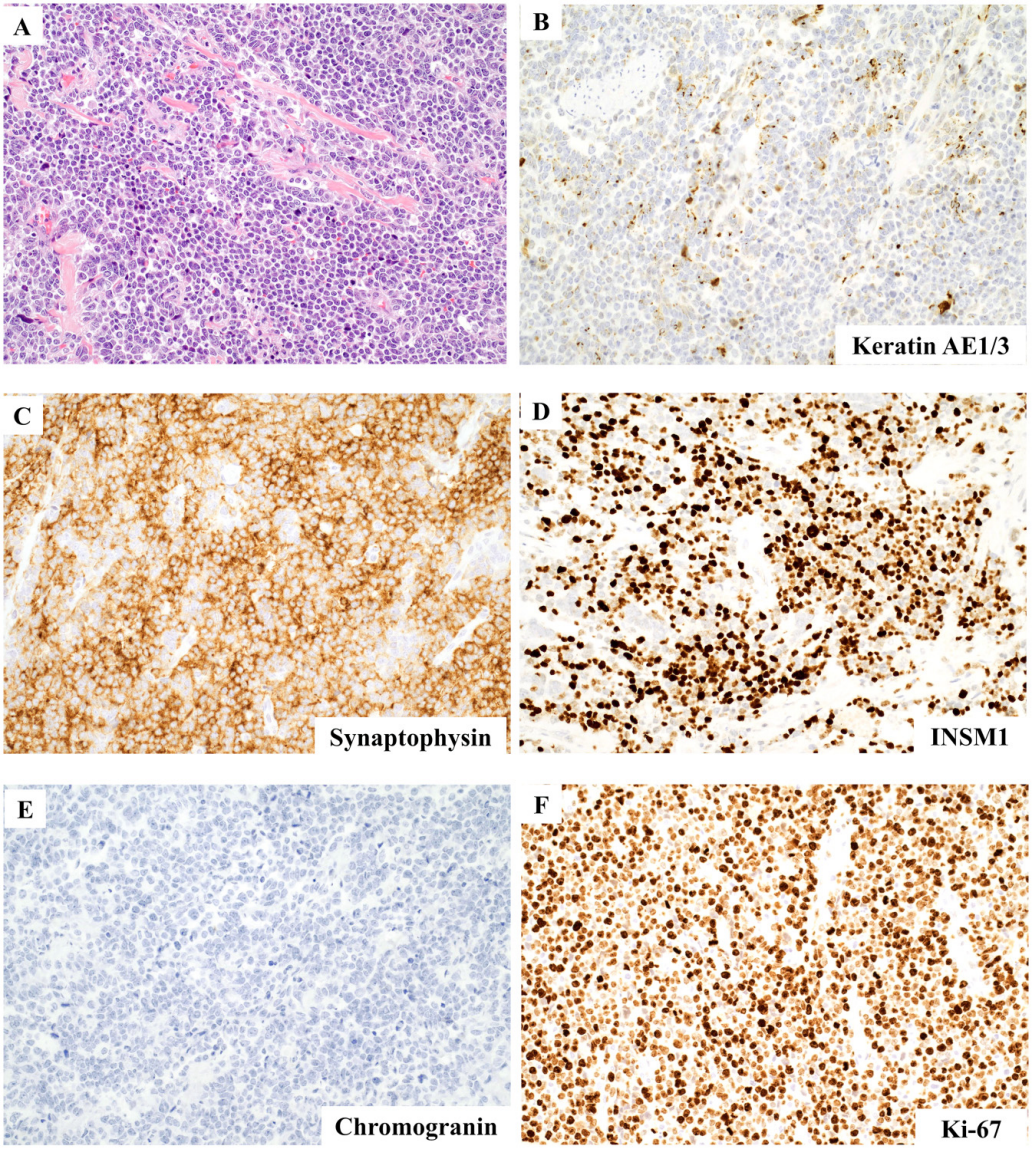
Microscopic examination and immunostains. (A) Large tumor iells (hematoxylin and eosin [H&E], 100×). (B) Dot-like staining pattern in keratin AE1/3 immunostain (200×). Diffuse positivity in synaptophysin (C) and INSM1 (D) immunostains (200×). Chromogranin immunostain is negative (E) (200×). Ki67 index is more than 90% (F) (200×).

The tumor was well-sampled with more than 25 tissue blocks submitted. Microscopic examination revealed the presence of a neoplasm comprising sheets of large cells in a semi-fascicular and pseudopapillary arrangement, with scant intervening stroma and extensive geographic necrosis (Figure 2). Scant cytoplasm, large pleomorphic nuclei with coarse chromatin, and occasionally prominent nucleoli were observed in the tumor cells. No apparent glandular components were identified. Immunostaining of the tumor cells revealed the following: strong and diffuse positivity for SATB2, INSM1, and synaptophysin immunostaining; patchy positivity for keratin AE1/3 (dot-like pattern), keratin CAM5.2, and keratin 20 immunostaining; and negative for CD45 (LCA), S100, SOX10, keratin 7, TTF1, CDX2, and chromogranin immunostaining, and mucicarmine special stain (Figure 3). The Ki67 index was >90%. These features are consistent with those of large-cell neuroendocrine carcinoma. Additionally, the possible appendix was grossly identified and submitted entirely, however, it was completely obliterated and destroyed by tumor cells. The tumor was classified as a colonic primary tumor, given its location in the cecum. Metastatic carcinoma cells were detected in three of the 23 lymph nodes. Evaluation of the colonic polyps revealed that they were tubulovillous adenomas with focal high-grade dysplasia and multiple tubular adenomas, consistent with the history of FAP. All resection margins were microscopically negative for carcinoma.

The expressions of the mismatch repair proteins MLH1, MSH2, MSH6, and PMS2 were intact by immunostains. Next-generation sequencing (FoundationOne^®^CDx 324 gene panel) revealed two mutations the *APC* gene, C1270* and H1490fs*23. Microsatellite stability was stable, with a low tumor mutational burden (two mutations per megabase). Pathogenic mutations in *PALB2* (T351fs*9) were present, which play critical roles in the DNA repair and tumor suppressor pathways. An activating mutation in *KRAS* (G12 V), a driver of oncogenesis, was observed. In addition, mutations in *MAP3K1* (E384*) and *NTRK3* (R731W), as well as a large deletion involving *RB1* (loss of exons 18-27), were detected, indicating a disruption in critical cell signaling and cycle regulatory pathways. Furthermore, a mutation in *TP53* (R196*) indicated impaired tumor suppression.

Adjuvant chemotherapy with cisplatin and etoposide was commenced postoperatively. Residual left colon was resected 12 months later. No pathological, clinical, or imaging evidence of recurrence was observed at the most recent follow-up visit 18 months after the initial right hemicolectomy.

## Discussion

Microscopic examination revealed a neoplasm comprising sheets of large cells with semi-fascicular and pseudopapillary arrangement and minimal intervening stroma in the present patient. Consistent with rapid tumor proliferation, extensive tumor necrosis possibly owing to the requirement exceeding the vascular supply was observed.^9^ The tumor cells exhibited scant cytoplasm, large pleomorphic nuclei with coarse chromatin, and occasional prominent nucleoli. These features are consistent with those of a high-grade malignancy with those of neuroendocrine differentiation.^10^ Differential diagnoses included poorly differentiated adenocarcinoma, high-grade lymphoma, melanoma, sarcoma, and mixed neuroendocrine-adenocarcinoma.^11,12^

Immunohistochemistry plays a critical role in narrowing down differential diagnoses. Positive keratin immunostaining ruled out the diagnoses of lymphoma, melanoma, and sarcoma. Positive INSM1 and synaptophysin immunostaining indicated neuroendocrine differentiation, ruling out the diagnosis of colorectal adenocarcinoma.^13^ Negative chromogranin staining, a classic marker for neuroendocrine cells that is less consistently positive in neuroendocrine carcinoma, was observed in the present patient. The expression of chromogranin may have been diminished or absent owing to the aggressive and poorly differentiated nature of the tumor. Synaptophysin is expressed more reliably in neuroendocrine carcinoma, providing stronger evidence of neuroendocrine differentiation in this patient.^14,15^ An extremely high Ki-67 index is a characteristic feature of poorly differentiated neuroendocrine carcinoma.^16^ Positive SATB2 immunostaining indicated colorectal origin, ruling out the diagnosis of a primary tumor from other sites.^17^ Positive staining for keratin 20, which is characteristic of colorectal carcinomas but uncommon in small cell neuroendocrine carcinoma and other neuroendocrine tumors outside the gastrointestinal tract, further supported the colorectal origin.^18,19^ Poorly-differentiated neuroendocrine carcinoma often exhibits a dot-like staining pattern of keratin AE1/3, which sets it apart from other carcinomas, which typically exhibit more diffuse cytoplasmic keratin expression.^20,21^ Keratin 7 negativity is atypical in pancreatic neuroendocrine tumors and other non-colorectal neoplasms. Thus, the presence of keratin 20 and the absence of keratin 7 further narrowed the differential diagnosis. CDX2 immunostaining, which is commonly observed in colorectal adenocarcinoma,^22^ was negative in the present patient. Negative immunostaining for S100, SOX10, and CD45 (LCA) further ruled out the diagnoses of melanoma and lymphoma.^23^ This immunohistochemical profile was highly suggestive of a large-cell neuroendocrine carcinoma of colorectal origin.

The mismatch repair proteins MLH1, MSH2, MSH6, and PMS2 demonstrated intact nuclear expression in the present patient, indicating a proficient mismatch repair status. Mismatch repair deficiency, particularly that of MLH1 or MSH2, indicates Lynch syndrome.^24,25^ However, the intact expression of all four mismatch repair proteins indicated the absence of an association with Lynch syndrome or microsatellite instability.

Estrella et al^26^ proposed that alterations in the APC/β-catenin pathway, central to FAP, may facilitate the development of well-differentiated neuroendocrine tumor components within intestinal adenomas. Compared with sporadic neuroendocrine tumors, which typically lack *APC* mutations, adenomas containing well-differentiated neuroendocrine tumor exhibited significantly higher nuclear β-catenin expression in their study. This increase in the β-catenin activity implies that *APC* mutations may drive tumorigenesis in the adenomatous and neuroendocrine components, suggesting a shared pathogenic origin within *APC*-mutated precursor cells. Thus, *APC* gene alterations in patients with FAP may contribute to conventional adenomas and neuroendocrine differentiation within these lesions, thereby broadening the potential scope of impact of *APC* on FAP-related neoplasia. Colonoscopy revealed multiple polyps (>100), including tubulovillous adenoma and tubular adenomas, along with the *APC* mutation identified by germline genetic test. These findings confirmed the diagnosis of FAP.^27^

*PALB2* encodes a key DNA repair protein that interacts with BRCA1/BRCA2 during homologous recombination. A *PALB2* (T351fs9) mutation was observed in the present study. This mutation results in a frameshift, leading to the formation of a truncated, non-functional protein that disrupts homologous recombination repair, thereby inducing genomic instability.^28,29^ *PALB2*-mutated cancers are sensitive to PARP inhibitors (eg, niraparib, olaparib, rucaparib, and talazoparib) owing to their synthetic lethality. These drugs target homologous recombination repair deficient tumors by inhibiting PARP, a key enzyme in single-stranded DNA break repair, leading to tumor cell death.^29–32^ *PALB2* mutations have been reported in 2.5% of patients with MSI-H colorectal cancer; however, to the best of our knowledge, *PALB2* mutations have not been documented in large-cell neuroendocrine carcinoma.^33^

Two *APC* mutations, C1270* and H1490fs23, were also identified in the tumor next generation sequencing test, indicating biallelic inactivation of *APC*, in which C1270* is of germline origin. *APC*, a tumor suppressor gene, regulates the WNT/β-catenin signaling pathway. These mutations result in the formation of truncated APC proteins that fail to regulate β-catenin degradation. This results in the activation of the WNT pathway, uncontrolled proliferation, inhibition of differentiation, and tumor progression. *APC* mutations often initiate colorectal cancer. Furthermore, they have been linked to aggressive phenotypes and poor outcomes when combined with other mutations.^34,35^ These mutations, observed in 59.5% of tumors, are common in colorectal neuroendocrine carcinoma.^36^

A *KRAS* (G12V) mutation, which represents an activating mutation in the *KRAS* oncogene, was detected in the present patient. This alteration drives tumor growth and contributes to resistance to certain therapies, particularly EGFR inhibitors. *KRAS* (G12V) mutations have been linked to aggressive tumor biology in patients with colorectal and pancreatic cancer, often correlating with shorter overall survival compared with other subtypes of *KRAS*.^37,38^ *KRAS* mutations, observed in 36.9% of tumors, are common in colorectal neuroendocrine carcinoma.^36^ Missense mutations, including G12D (G12A, G12C, G12R, G12V) and G13D, are the most prevalent *KRAS* mutations in colorectal neuroendocrine carcinoma.^36^ These mutations are mutually exclusive to *BRAF* mutations. The presence of *KRAS* mutations is associated with poorer survival outcomes in patients with colorectal neuroendocrine carcinoma.^36^

A *MAP3K1* (E384*) mutation, representing a truncating mutation in the MAP kinase signaling cascade, was also observed in the present patient. *MAP3K1* mutations may disrupt cell growth and apoptosis regulation, thereby contributing to tumor progression.^39^ Alterations in *MAP3K1*, which promote survival pathways, have been linked to chemoresistance.^40^ *MAP3K1* mutations were detected in two out of the 68 samples of colorectal neuroendocrine carcinoma, corresponding to a mutation rate of 2.9%.^36^

*NTRK3* encodes a receptor tyrosine kinase that regulates cellular proliferation, survival, and differentiation through the PI3 K/AKT, MAPK/ERK, and JAK/STAT pathways. An *NTRK3* R731 W mutation was also observed in the present patient. R731 W mutations may lead to constitutive activation of downstream signaling, thereby promoting oncogenic processes such as uncontrolled proliferation and resistance to apoptosis.^41^ *NTRK3* mutations, detected in 31% of tumors, are frequently observed in large-cell neuroendocrine carcinoma. *NTRK* mutations were observed in nine of the 95 pulmonary neuroendocrine tumor, exclusively within large-cell neuroendocrine carcinoma, in a study of 538 lung carcinoma samples. However, *NTRK* mutations were not observed in the 443 non-small cell lung carcinoma samples without neuroendocrine differentiation, reinforcing the strong association between *NTRK* alterations and the large-cell neuroendocrine carcinoma histotype.^42^

An *RB1* (loss of exons 18-27) mutation affecting *RB1*, which encodes the retinoblastoma protein (pRb), was identified in the present patient. *RB1* is a crucial tumor suppressor that regulates the progression of the cell cycle by inhibiting the transition from the G1 phase to the S phase. Loss of exons 18–27 disrupts the interaction of pRb with E2F transcription factors, resulting in uncontrolled cell division, genomic instability, and tumor progression.^43,44^ *RB1* mutations were detected in 26% of lung large-cell neuroendocrine carcinomas. Another study reported a mutation frequency of 31%, with an additional 7% of tumors exhibiting *RB1* loss and 40% of tumors exhibiting pRb protein loss. These findings indicate that multiple mechanisms may contribute to the inactivation of *RB1*.^45^ Experimental models have revealed that neuroendocrine cells may be particularly susceptible to *RB1* loss. Thus, *RB1* inactivation plays a key role in the development of neuroendocrine carcinoma.^45^

A *TP53* (R196*) mutation resulted in a truncated, nonfunctional p53 protein in the present patient. *TP53*, a crucial tumor suppressor, regulates the cell cycle, DNA repair, and apoptosis. Loss-of-function mutations such as R196* impair these mechanisms, resulting in uncontrolled proliferation, genomic instability, and increased tumorigenicity. *TP53* mutations have been implicated in aggressive tumor behavior and poor overall survival across multiple types of cancer, including colorectal cancer.^46,47^ *TP53* mutations have been observed in 65.5% of colorectal neuroendocrine carcinomas, with missense mutations being observed most frequently.^36^ Similarly, *TP53* mutations, identified in 78% of tumors in one study,^45^ are highly prevalent in lung large-cell neuroendocrine carcinoma. Thus, *TP53* is considered the most frequently altered gene in large-cell neuroendocrine carcinoma.^48^

Detweiler et al^8^ also reported a colorectal neuroendocrine carcinoma in a patient with a history of FAP. Comparison of the findings of this report with the findings of their report revealed notable differences in terms of the immunohistochemical profiles, differentiation, tumor grade, and clinical presentation. The tumor described in the present patient exhibited a distinct immunohistochemical profile. Furthermore, the tumor stained positively for INSM1, synaptophysin, SATB2, keratin 20, keratin AE1/3, and keratin CAM5.2 and negative for chromogranin, CDX2, and keratin 7. These findings confirmed the diagnosis of large-cell neuroendocrine carcinoma. In contrast, the tumor described in the report by Detweiler et al exhibited mixed histological differentiation, including glandular and squamous components arising from an adenomatous polyp. The Ki-67 index was >90% in both tumors, indicating the highly aggressive nature of both tumors. Extensive metastasis to the lymph nodes and liver at the time of diagnosis was reported by Detweiler et al, whereas only lymph node metastasis was observed in the present patient. These differences underscore the variability in the presentation and characteristics of neuroendocrine carcinoma associated with FAP.

## Conclusion

This case report described a rare presentation of colorectal large-cell neuroendocrine carcinoma in a patient with FAP, which has been reported only once in the literature. The association between *APC* mutations and neuroendocrine tumorigenesis is poorly understood. This case report contributes to the limited literature on colorectal neuroendocrine carcinoma in patients with FAP, underscoring the requirement for further research on the molecular mechanisms underlying this association.
